# Editorial of the Special Issue “Neurobiological Mechanisms Implicated in Stress-Related Psychiatric Disorders”

**DOI:** 10.3390/ijms24097856

**Published:** 2023-04-26

**Authors:** Zoya Marinova

**Affiliations:** Ronin Institute for Independent Scholarship, Montclair, NJ 07043, USA; zoya.marinova@ronininstitute.org

Mental disorders may seriously impair the quality of life of affected individuals and cause a significant public health burden. For example, mental disorders have been responsible for over 14% of age-standardized years lived with a disability during the past three decades [1]. Further, across the lifespan, the relative disease burden of different conditions affecting the central nervous system (CNS) may vary, with autism spectrum disorder and idiopathic intellectual disability being prominent during childhood, and depressive, anxiety, and psychotic disorders playing a key role during adulthood.

Stress has been identified as one of the risk factors for impaired mental health. Moreover, the nature, timing, and duration of trauma are apparently important regarding its effect. Thus, the association between childhood trauma and risk of mental illness later in life has been clearly established. In particular, childhood maltreatment has been correlated with increased odds of mood, anxiety, and drug abuse disorders (ORs = 2.1–4.1), and this association has been confirmed both by prospective and retrospective investigations [2]. Within the context of childhood adversities, family dysfunction and abuse have been identified as strong predictors of different mental disorders across life stages [3]. However, trauma during the later stages of life may also increase the risk of mental illness. Natural disasters, industrial disasters, wars and conflict, climate change, and forced migration have also been implicated as social determinants of mental disorders [4].

The molecular mechanisms, via which stress can affect mental health and health overall, have been only partially elucidated, and they are multifaceted, including both direct biological effects and changes in health-related behaviors. However, additional research into the mechanisms underlying the effects of stress on the risk of mental illness is required to fully elucidate the underlying mechanisms and identify novel therapeutic targets and biomarkers.

The current Special Issue aimed to further investigate the molecular mechanisms via which stress affects the risk of mental illness. Several of the articles focused on the significance of hypothalamic–pituitary–adrenal (HPA) axis dysregulation in stress and mental illness. Since retinoic acid has been implicated in stress-related disorders, Lin et al. analyzed the role of endogenous cellular retinoic acid-binding protein 1 (Crabp1) using both a knockout mouse model and an in vitro cell culture model. They found that retinoic acid and stress can elevate Crabp1 levels, which increases the expression of FKBP prolyl isomerase 5 (FKBP5), inhibits glucocorticoid signaling, desensitizes the feedback inhibition of the HPA axis, and elevates the risk of stress-related disorders [5]. 

A dysfunction of the HPA axis may also be implicated in the increased comorbidity between infections with the human immunodeficiency virus (HIV) and stress-related mental disorders. In this context, Salahuddin et al. examined the role of HPA dysregulation in the interactions of the key HIV viral protein trans-activator of transcription (Tat) with oxycodone using Tat-expressing mice. Acutely administered oxycodone interacted with HIV-1 Tat to potentiate anxiety-like and psychomotor behaviors, whereas repeated oxycodone exposure sensitized the HPA stress response and stress-related psychomotor behaviors. Further, oxycodone-mediated behaviors were ameliorated by a blockade of the glucocorticoid receptors or corticotrophin-releasing factor (CRF) receptors. The findings suggested that Tat can dysregulate the HPA axis, implying that it might increase the risk of stress-related substance use and mood disorders [6]. One of the neuromodulators involved in the negative feedback mechanisms of the HPA axis is the neurosteroid allopregnanolone. Almeida et al. reviewed the role of allopregnanolone both as a positive γ-amino butyric acid (GABA)-ergic neuromodulator and an inhibitor of the HPA axis. Further, they discussed stress-induced changes in allopregnanolone and its potential as a treatment target and biomarker [7].

The role of inflammation in mental disorders, including major depressive disorder, has also emerged. Two of the articles in this Special Issue focused on the role of inflammation in depression. Suneson et al. reviewed the evidence identifying inflammatory depression as a distinct depression subtype. They discussed upstream mechanisms promoting low-grade inflammation, including dysbiosis and an increased dietary omega-6/omega-3 ratio. In addition, the authors reviewed downstream mechanisms potentially linking inflammation and depressive symptoms, such as changes in dopaminergic signaling and tryptophan metabolism. Finally, they discussed several possible non-pharmacological interventions for inflammatory depression, including probiotics, omega-3 fatty acids, and physical exercise [8]. Won et al. reviewed melatonin’s inhibitory effects on inflammatory responses, including neuroinflammation, as well as its circadian rhythm-modulating, neuroprotective, and anti-depressant activities. They also discussed the significance of future research to investigate the therapeutic potential of melatonin in patients with major depressive disorder or neurodegenerative disorders [9].

As there is a need to identify reliable biomarkers for stress-related mental disorders, a study by Hernandez-Baixauli et al. searched for candidate biomarkers. It used omics profiling of plasma and urine metabolites in combination with metagenomics in a chronic, unpredictable, mild stress animal model. The authors discovered a signature including eight plasma metabolites, six urine metabolites, and five microbes. In particular, cholesterol, alpha-ketoglutarate, malic acid, threonic acid, and succinic acid were identified as key metabolites that could be potential biomarkers of early stages of stress in the plasma metabolome [10]. 

Stress may affect not only mental but also physical health. Wuertz-Kozak et al. evaluated the effect of early life stress on bone mineral density (BMD), bone microarchitecture, metabolic parameters, and neuronal stress mediators. In mice exposed to early stress, the authors detected decreased nerve growth factor (NGF), neuropeptide Y receptor 1 (NPYR1), tachykinin receptor 1 (TACR1), and vasoactive intestinal peptide receptor 1 (VIPR1) levels. Moreover, the levels of C-terminal telopeptide of type I collagen (CTX-I) were increased, and bone innervation density was enhanced. These findings were indicative of a milieu favoring catabolic bone turnover. In patients with depression and a history of severe childhood stress exposure, a BMD reduction was observed. In addition, the severity and type of childhood stress also influenced its effect on serum bone markers, suggesting the need for further, more personalized investigations [11]. Notably, cognitive problems may be observed in association with mental illness. Morozova et al. reviewed data on the prevalence of cognitive problems in patients with disorders of the CNS, as well as the associated neurobiological mechanisms and effects on quality of life [12].

Imaging studies can provide important clues regarding the neurocircuitry involved in mental disorders. Kim et al. reviewed neuroimaging reports on generalized anxiety disorder (GAD). Their findings confirmed various degrees of abnormal blood oxygenation level-dependent (BOLD) responsivity in the corticolimbic circuitry of patients with GAD, including the amygdala, prefrontal and anterior cingulate cortex, hippocampus, and insula [13].

The functional role of the metabotropic glutamate type 5 receptor (mGluR5) in the stress response was investigated by Zangrandi et al. using a mouse model with conditional mGluR5 knockout in dopamine D1 receptor-expressing neurons. Upon exposure to acute stress, there were changes in the coping mechanisms of mice with conditional mGluR5-D1 knockout. Under the condition of inescapable stress, these mice adopted an enhanced passive coping strategy, whereas, under the condition of acute escapable stress, they adopted an enhanced active coping strategy. These findings provided insights into the role of mGluR5, D1 receptor-expressing neurons, and their functional integration as key mediators of behavioral response to stress [14].

The involvement of serotonin/serotonin 1A (5-HT1A) receptor signaling in the endothelial expression of claudin-5 was evaluated by Sugimoto et al. They found that the 5-HT1A receptor is expressed in brain microvascular endothelial cells (BMVECs) and mural cells in post-mortem human cortex. In a two-dimensional co-culture of a human brain-derived pericyte cell line and human primary BMVECs, an endothelial cell-pericyte tube-like structure was formed. In addition, serotonin/5-HT1A receptor signaling promoted endothelial claudin-5 expression in the co-culture. These findings supported the role of serotonin/5-HT1A receptor signaling in the regulation of the blood–brain barrier in a region-specific manner [15].

Duda et al. investigated the interactions of fructose 1,6-bisphosphatase 2 (Fbp2), an enzyme implicated in synaptic plasticity and mitochondrial function, with Co^2+^, which, at excessive concentrations, has been associated with CNS pathology. The authors found that Co^2+^ blocked the transition of Fbp2 to the canonical T-state and instead triggered a non-canonical, partially active T-state of Fbp2. In this state, Fbp2 could interact with its binding partners, including Ca^2+^/calmodulin-dependent protein kinase 2α (Camk2α), and could form an Fbp2-Camk2α complex, facilitating the autoactivation of Camk2α and synaptic plasticity [16].

Stress has been implicated in the vulnerability to mental illness. This Special Issue published articles aiming to provide further insights into underlying neurobiological mechanisms. They highlighted the significance of the HPA axis, inflammation, and serotonergic, dopaminergic, and glutaminergic signaling in the relationship between stress and mental disorders. In addition, they contributed data on potential stress biomarkers and the interactions between stress, mental, and physical health.
